# Influence of Feeding Linseed on SCD Activity in Grazing Goat Mammary Glands

**DOI:** 10.3390/ani9100786

**Published:** 2019-10-11

**Authors:** Raffaella Tudisco, Biagina Chiofalo, Vittorio Lo Presti, Valeria Maria Morittu, Giuseppe Moniello, Micaela Grossi, Nadia Musco, Raffaella Grazioli, Vincenzo Mastellone, Pietro Lombardi, Federico Infascelli

**Affiliations:** 1 Department of Veterinary Medicine and Animal Production, University of Napoli Federico II, 80100 Napoli, Italy; tudisco@unina.it (R.T.); pietro.lombardi@unina.it (P.L.); micaelagrossi@tiscali.it (M.G.); raffaella.grazioli@unina.it (R.G.); vincenzo.mastellone@unina.it (V.M.); federico.infascelli@unina.it (F.I.); 2 Department of Veterinary Science, University of Messina, 98122 Messina, Italy; bchiofal@unime.it (B.C.); vittorio.lopresti@unime.it (V.L.P.); 3 Department of Health Sciences, Magna Graecia University of Catanzaro, 88100 Catanzaro, Italy; morittu@unicz.it; 4 Department of Veterinary Medicine, University of Sassari, 07100 Sassari, Italy; moniello@uniss.it

**Keywords:** goat, stearoyl-CoA desaturase, CLA, linseed

## Abstract

**Simple Summary:**

The effect of linseed feeding on stearoyl-CoA desaturase (SCD) activity was studied in dairy goats. SCD acts on the synthesis of milk conjugated linoleic acids (CLAs), considered highly important for human health. Linseed feeding significantly changed SCD activity, milk fat, and fatty acid profile; in particular, CLAs were higher in treated animals with potential benefits for human health. Because of the increasing care of consumers for the healthy aspects of foods, results increase the knowledge of beneficial effects of goat milk due to animal nutrition.

**Abstract:**

The effects of linseed feeding on the stearoyl-CoA desaturase (SCD) activity were evaluated on grazing dairy goats divided into two homogeneous groups (C, control, and L, treated) fed the same amount of concentrate which, for group L was supplemented with linseed. Milk yield was unaffected by the treatment. Group L showed significantly higher milk fat (4.10% vs 2.94%, *p* < 0.01) than group S. Within milk fatty acids, group C showed significantly higher levels of saturated fatty acids and lower values of mono-unsaturated and polyunsaturated fatty acids. In group L, total CLAs were higher than in group S (0.646% vs 0.311%; *p* < 0.01) mainly because of the differences in CLA cis9 trans 11 (0.623% vs 0.304%; *p* < 0.01). In treated animals, SCD activity, measured as cis9 C14:1/C14:0, was lower than in the control group, mainly in July and August.

## 1. Introduction

The inclusion of polyunsaturated fatty acids (PUFAs) in the diet of ruminants has been associated with the increase of these fatty acids in milk with, particularly referring to omega 3 PUFA, potential advantages for human health [1]. In addition, incomplete rumen biohydrogenation of linoleic acid (LA, C18:2, omega 6) and α-linolenic acid (ALA, C18:3, omega 3) involves an increase of conjugated linoleic acids (CLAs) isomers [2] which are recognized as anticarcinogenic, anti-atherosclerotic and immunomodulator [3]. CLAs come also from the activity of the mammary gland Stearoyl-CoA Desaturase (SCD) on trans-11 C18:1 (TVA, trans vaccenic acid), an intermediate of rumen PUFAs biohydrogenation. Furthermore, SCD transforms several saturated fatty acids (SFAs), particularly myristic (C14:0), palmitic (C16:0) and stearic (C18:0) in monounsaturated fatty acids (MUFAs) by a cis double bond between carbons 9 and 10 [4]; thus, according to Lock and Garnsworthy [5], SCD activity can be measured by comparing the product/substrate ratios of those fatty acids. In recent years, several studies focusing on dietary source of PUFAs able to improve the milk nutritional characteristics have been conducted [6,7,8,9,10,11]. Among them, much attention is paid to linseed (*Linum usitatissimum* L.) containing a high level of ALA (50% to 60% of total fatty acids, FA) [12] and lower concentration of LA and SFA compared to soybean, cottonseed, corn, and sunflowers [13]. Supplementing goat diet with 30 g/day of linseed, Gomez Cortes et al. [14] found a significant increase of vaccenic acid and ALA acid in milk. Similarly, Nudda et al. [15] reported an increase in vaccenic acid, PUFA, and CLA, adding 180 g/day of linseed to the Saanen goat diets consisting of both pasture and hay while Renna et al. [16] found higher values of ALA with a reduction of omega 6/omega 3 ratio and of SFA in milk from Saanen goats fed diet supplemented with 80 g/day of linseeds. Finally, the partial substitution of soybean with linseed in the diet of dairy goats resulted in an increase in milk branched fatty acids, total omega 3, and ALA [17]. Due to the increasing interest of consumers for the food nutritional characteristics, particularly for fatty acids profile, and taking into account the pivotal role of SCD in the CLA synthesis, this research aimed to evaluate the effect of feeding linseed to grazing goats on the activity of this enzyme in the mammary gland. 

## 2. Materials and Methods

### 2.1. Animals, Diets, and Management

The trial was performed on 16 pregnant Cilentana dairy goats (50.0 ± 2.0 kg body weight) in a farm located at Casaletto Spartano, Salerno province (Italy), at 832 m a.s.l., according to the Animal Welfare and Good Clinical Practice (Directive 2010/63/EU) and after the approval of the local Bioethics Committee (protocol number: PG/2019/0070006). The goats were fed ad libitum oat hay and 100-200 and 300 g/head/day of concentrate 45-30 and 15 days before the kidding (first week of March), respectively. Successively, they were equally divided into two groups (C, control, and L, linseed), homogeneous for parity (3rd) and milk yield at the previous lactation (1300 ± 108 g/h/day), having free access to pasture (9.00 am to 4.00 pm), constituted by 60% Leguminosae (Trifolium alexandrinum, Vicia spp.) and 40% Graminae (Bromus catharticus, Festuca arundinacea, Lolium perenne). Group C received a supplement of 400 g/head of concentrate (CC), while group L received 400 g/head of an isoprotein concentrate (LC) characterized by the addition of linseed (in ratio of 20% as fed) among the ingredients. 

Samples of pasture were monthly collected from three different areas (2.5 square meters each) at no less than 3 cm from the ground. After weighing, samples were air-oven dried at 65 °C, milled through a 1 mm screen and stored. The chemical composition of feeds was analyzed according to Van Soest et al. [18] and AOAC [19] while the net energy was calculated as suggested by INRA [20]. 

Pasture and concentrates fatty acids (FA) profiles were analyzed as follows: the total fat was extracted according to Folch et al. [21]; successively, the base-catalysed procedure of Christie [22], modificated by Chouinard et al. [23], was adopted for the FA transmethylation. The methyl esters were quantified by gas chromatograph (ThermoQuest 8000TOP gas chromatograph, equipped with flame ionization detector; ThermoElectron Corporation, Rodano, Milan, Italy) equipped with a CP-SIL 88 fused silica capillary column [100 m 0.25 mm (internal diameter) with 0.2-lm film thickness; Varian, Walnut Creek, CA, USA] set according to Tudisco et al. [24]. FA peaks were identified using pure methyl ester external standards (Larodan Fine Chemicals, AB, Limhamnsgardens Malmo, Sweden) and comparing the retention times with those of the standard mixture. 

### 2.2. Milk Analysis

From days 0 to 60 milk was totally suckled by kids; successively, daily milk yield was recorded, and representative milk samples (weighted average of the two daily milkings), for a total of 5 sampling, were monthly analysed for fat, protein, and lactose concentration by the infrared method (Milkoscan 133B, Foss Matic, Hillerod, Denmark). In addition, total fat of milk samples was separated using a mixture of hexane/isopropane (3/2 v/v), as described by Hara and Radin [25]. FA transmethylation and quantification were performed as above; additional standards for CLA isomers were from Larodan (Larodan Fine Chemicals, AB, Limhamnsgardens Malmo, Sweden). 

The SCD activity index was calculated by the following product/substrate ratios: C14:1/C14:0, C16:1/C16:0, C18:1/C18:0 and C18:2 c9 t11/C18:1 t11, as suggested by Lock and Garnsworthy [5].

### 2.3. Statistical Analysis

Data were analysed using the MIXED procedure of the JMP software (SAS Institute, NC, USA, 2000) for repeated measures over time. The goat was considered as the experimental unit. 

The following model was used:

Yijk = µ + DTi + G(i)j + STk + (DT + ST) ik + Ɛijk(1)

where Yijk = mean of response variable, µ = population mean, DTi = effect of the dietary treatment (i = 2; C and L), G(i)j = random effect of goat within the treatments, STk = effect of sampling time (k = 5; May, June, July, August, September), (DT × ST) ik = fixed effect of interaction between dietary treatment and sampling time, and εijk = experimental error. 

The comparison among the mean values was performed by using Tukey’s test. Differences were considered statistically significant at *p* < 0.05.

## 3. Results 

Goats body weights (BW) did not differ between the groups along the trial and no refusals were found. In Table 1, feeds chemical composition, nutritive value, and fatty acids profiles are reported. The two concentrates showed 1.05 vs 1.16 forage units for milk production (UFL)/kg dry matter, DM nutritive value for CC and LC, respectively and similar crude protein concentration (CP % DM: 18.0 vs 18.2 for CC and LC, respectively) while pasture CP (CP/DM: 16.4%) and nutritive value (0.77 UFL/kg DM) were comparable with those reported in previous trials performed on the same area [24,26,27,28]. The fatty acids profiles of concentrates were different: CC showed slightly higher saturated (SFA) and monounsaturated (MUFA) and lower polyunsaturated (PUFA) fatty acids percentage than LC. LA was higher in CC while ALA was considerably higher in LC due to linseed being among the ingredients. Pasture showed remarkably high PUFA with ALA, almost double than LA. 

Milk yield and fat (Table 2) were significantly higher in group L as mean values (1372.7 vs 1133.6 g/day; 4.38% vs 4.20%; *p* < 0.01 for group L and C respectively) as well as along the trial. Protein and lactose were unaffected by the treatment, even if the sampling effect was significant (*p* < 0.01) for lactose.

In Table 3, the milk fatty acids profile and the product/substrate ratios used to measure SCD activity are reported. Group L showed significantly lower average level of C11:0 (0.130 vs 0.158; P < 0.05), C14:0 (8.74 vs 10.24; *p* < 0.01), C14:1 (0.24 vs 0.33; *p* < 0.01), C15:0 (0.62 vs 0.82; *p* < 0.01), C15:1 (0.14 vs 0.20; P < 0.05), C16:0 (23.50 vs 28.53; *p* < 0.01), C16:1 (0.33 vs 0.42; *p* < 0.01), C17:0 (0.55 vs 0.70; *p* < 0.01), C17:1 (0.14 vs 0.22; *p* < 0.01), C20:0 (0.50 vs 0.55; *p* < 0.05) and of total SFA (69.12 vs 73.29; *p* < 0.01) and significantly higher of C18:0 (16.31 vs 13.42; P < 0.01), C18:1 omega 9 (4.79 vs 2.09; P < 0.01), C18:2 trans9 trans12 omega 6 (0.55 vs 0.19; *p* < 0.01), total MUFA (26.62 vs 23.44; *p* < 0.01), total PUFA (4.27 vs 3.29; *p* < 0.01), total PUFA omega 6 (2.22 vs 1.79; *p* < 001), cis9 trans11 CLA (0.63 vs 0.34; *p* < 0.05) and total CLA (0.71 vs 0.43; *p* < 0.05). Concerning the SCD activity, except C16:1/C16:0 the other product/substrate ratios were significantly different between the groups: C18:1/C18:0 and cis9 trans11 CLA/C18:1 trans 11 were higher for group L while C14:1/C14:0 was higher for group C.

## 4. Discussion 

The supplementation of linseed in group L resulted in increasing milk yield according to the results reported by Nudda et al. [29] in Saanen goats. On the contrary, Nudda et al. [15], Bernard et al. [8] and Almeida et al. [30] did not register a similar effect in lactating goats fed with linseed. It should be underlined that for both the groups of this study the energy requirements (ER) were satisfied. Indeed, pasture dry matter intake of goats from local genotypes bred in southern Italy is 20 g/kg BW [31], while ER are 0.0365 UFL/kg metabolic weight (maintenance) plus 0.41 UFL/kg 4% fat corrected milk. Thus, goats weighing 50 kg BW ingested 1 kg DM of pasture, which account to 0.77 UFL while ER were 1.25 UFL (0.69 for the maintenance plus 0.56 MUF for milk yield). The concentrates covered the deficit (0.4 kg are more than 0.4 UFL). Milk fat was higher in goats fed linseed, in contrast to the results of Loor et al. [32] and Almeida et al. [30]. These authors found no effect of dietary treatment on milk fat percentage as a consequence of the physiological ruminal fermentation patterns [33]. Our results agree with those obtained in goats by Renna et al. [16] and in dairy cows by Di Trana et al. [34] Palmquist and Conrad [35]; according to these last authors, supplementing fat to the diet does not depress feed intake and milk fat. Milk protein was unaffected by the treatment, as reported also by Chilliard et al. [36] due to the fact that fat supplementation to the diet did not affect energy intake, which is the most important nutritional factor affecting milk protein. For the same reason, milk lactose was not affected by dietary treatment. Milk from goats fed linseed showed significantly (*p* < 0.01) lower SFA and significantly (*p* < 0.01) higher MUFAs and PUFAs, according to the results of Nudda et al. [15] in goats and Caroprese et al. [37] in sheep. In our study, omega 6 PUFA were significantly (*p* < 0.01) higher in treated group while omega 3 PUFA were unaffected by dietary fat supplementation. 

Concerning milk CLAs, in ruminants they originate in the mammary gland from rumen biohydrogenation and endogenous synthesis by SCD activity on trans11 C18:1 (TVA), that is an intermediate product of PUFA biohydrogenation [38].

In the treated group, milk CLAs were significantly (*p* < 0.01) higher than in the control group. Since both groups were grazing, this result has to be attributed only to the fatty acid profile of concentrates (Table 1): LC had higher ALA level than CC, where LA was more represented. Thus, the CLAs were mainly synthesized from ALA than LA. On the other hand, this could also explain the lack of difference of ALA in milk between the groups. Similar increases of milk CLA are reported by Martinez Marín et al. [39] and Mele et al. [40] respectively in goats and sheep fed linseed. 

SCD activity in the mammary gland of ruminants is calculated by comparing the product/substrate ratios of some fatty acids, cis9 C14:1, cis9 C16:1, cis9 C18:1 and CLA, produced from C14:0, C16:0, C18:0 and trans11 C18:1, respectively. According to Lock and Garnsworthy [5], the best indicator of SCD activity is the cis9 C14:1/C14:0 ratio because all of the C14:0 in milk fat is produced via de novo synthesis in the mammary gland; consequently, desaturation is the only source of C14:1. Increasing cis9 C14:1/C14:0 ratio values would indicate an increase of SCD activity.

**Figure 1:**
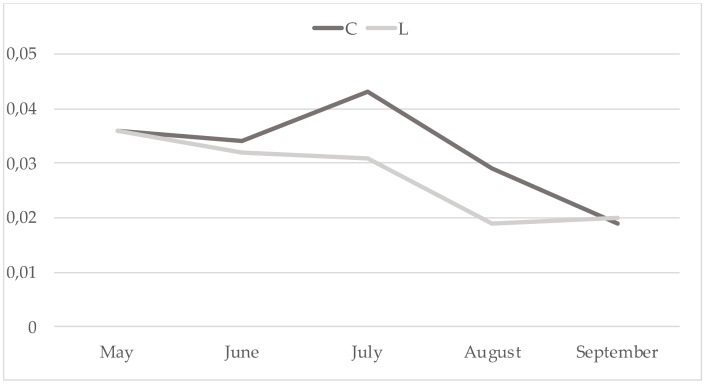
Trend of cis 9 C14:1/C14:0 ratio (SEM = 0.0008).

**Figure 2:**
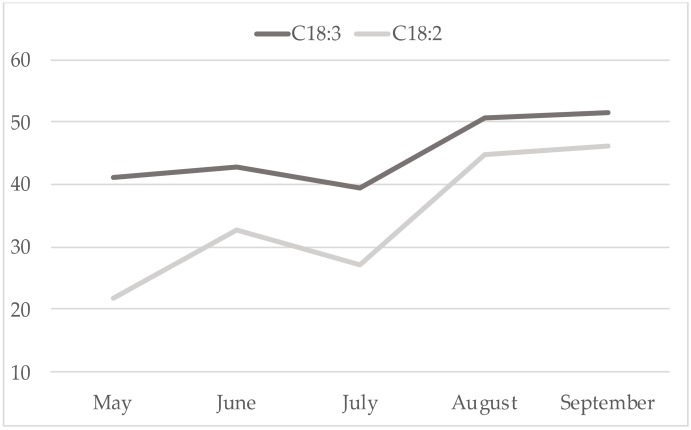
Trend of ALA and LA of the pasture (% total FA).

**Figure 3:**
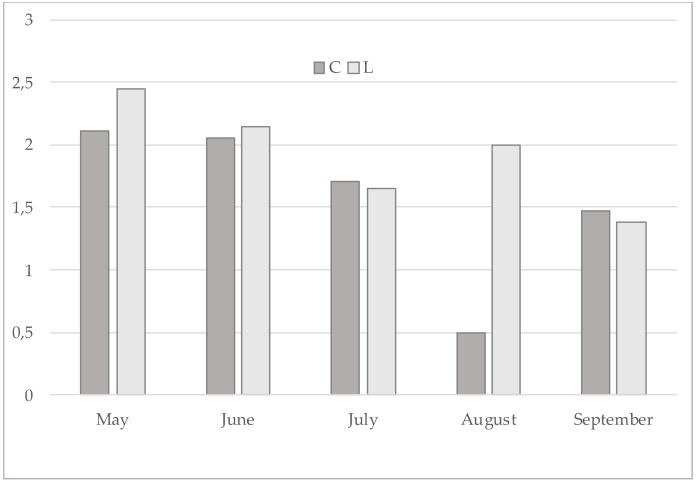
CLAs content (% total FA) in control and treated group along the trial.

Noteworthy, in this study cis9 C14:1/C14:0 was significantly (*p* < 0.01) lower in the treated group. Analysing this parameter along the trial (Figure 1), the phenomenon could be attributed particularly to the milk samples effected in July and August when, in the area of the research, the pasture is in vegetative stasis with lower PUFA concentration (Figure 2). Therefore, in that period the contribute of concentrate was higher: LC had higher PUFAs (% total fatty acids: 61.4 vs 56.6, for LC and CC, respectively), confirming their down-regulation effect on mammary gland SCD activity [41]. In contrast, dietary PUFAs, being substrates for rumen biohydrogenation, determined an increase of the other product/substrate ratios in treated group. Sunflower seed oil [6] and linseed oil [7] administration in goats’ maize silage-based diets had no effects on SCD activity, while in a grass hay-based diet they just reduced it [6]. A similar effect was described by Bernard et al. [8] adding soya beans to alpha-alpha hay-based diets. Thereafter, the same authors [42] suggested that the interaction among different ingredients of the diet is also of great importance because both rumen-bypass PUFA and bio-hydrogenation intermediates are able to inhibit SCD acting by transcriptional or post-transcriptional mechanisms. Interestingly, milk CLAs content was higher in August in the treated group (Figure 3), thus suggesting that PUFAs induce a downregulation of SCD but exerts a positive effect on CLAs [43].

## 5. Conclusions

The supplementation of linseed in the diet of grazing goats did not affect milk yield. On the contrary, milk fat increased with the treatment. This result is interesting because in the area of our research, goat milk is used for cheese-making. Moreover, feeding linseed to grazing animals resulted in an improvement of the nutritional characteristics of milk, particularly for CLAs, whose increase is known to be beneficial for human health. Indeed, the best indicator of SCD activity was lower in the treated group, thus suggesting that animal feeding plays a pivotal role for CLAs synthesis. 

## Figures and Tables

**Table 1:** Feeds chemical composition (g/kg DM), nutritive value and FA profile (% total FA).

Chemical Characteristics	CC*	LC**	Pasture
Crude protein	180.0	182.0	164.0
Ether extract	30.0	71.0	21.0
NDF	264.0	270.0	489.0
ADF	101.0	105.0	343.0
ADL	28.0	29.0	47.0
UFL***/kg DM	1.05	1.16	0.77
**Fatty acid profile (% of total FA)**			
SFA	29.0	26.6	18.2
MUFA	14.4	12.0	6.0
PUFA	56.6	61.4	75.8
C18:2	46.0	35.8	28.1
C18:3	3.2	18.9	43.0

*CC (concentrate for group C) (% as fed): soft wheat bran 26.6; corn meal 15.0; sunflower meal 14.5; dried pulp beet 12.0; faba bean 10.6; corn gluten feed 7.0; dried citrus pulp 6.5; molasses 5.6; vitamin-mineral premix 2.2. **CL (concentrate for group L) (% as fed): soft wheat bran 30.0; corn meal 23.0; linseed 20.0; dried citrus pulp 10.0; dried pulp beet 8.0; corn gluten feed 7.0; vitamin-mineral premix 2.0. SFA, saturated fatty acids; MUFA, monounsaturated fatty acids; PUFA, polyunsaturated fatty acids. ***UFL (unité fourragère lait) = feed unit for lactation where 1 UFL equals 7.11 MJ of net energy.

**Table 2:** Milk yield (g/head/day) and chemical composition (%).

Milk Characteristics	Yield		Fat		Protein		Lactose	
Group	C	L	C	L	C	L	C	L
	1133.6	1372.7	4.20	4.38	3.53	3.58	4.00	4.02
**Group effect**	**		**		NS		NS	
**Sampling effect**	**		**		NS		**	
**G x S**	NS		NS		NS		NS	
**SEM**	300.1		0.621		0.246		0.24	

**, *p* < 0.01; NS, Not Significant. SEM, standard error of mean.

**Table 3:** Milk fatty acid profile (% total fatty acids).

Fatty Acids Profile	C	L	Group Effect	Sampling Effect	G x S	SEM
C4:0	1.42	1.53	*	*	NS	0.225
C6:0	1.91	2.07	NS	*	NS	0.23
C8:0	2.53	2.67	NS	**	NS	0.31
C10:0	9.00	8.76	NS	**	NS	0.96
C11:0	0.158	0.130	*	**	**	0.03
C12:0	3.57	3.26	NS	**	NS	0.53
C14:0	10.24	8.74	**	**	NS	0.83
C14:1 cis 9	0.33	0.24	**	**	**	0.04
C15:0	0.82	0.62	**	**	**	0.07
C15:1	0.20	0.14	*	**	**	0.02
C16:0	28.53	23.50	**	**	NS	1.83
C16:1 cis9	0.42	0.33	**	**	**	0.10
C17:0	0.70	0.55	**	**	**	0.12
C17:1	0.22	0.14	**	NS	NS	0.03
C18:0	13.42	16.31	**	**	NS	1.61
C18:1 cis 9	2.09	4.79	**	NS	NS	0.61
C18:1 trans 11	20.17	20.97	NS	*	NS	1.92
LA, C18:2 trans 9 trans 12 omega 6	0.19	0.55	**	*	**	0.15
C18:2 cis 9 cis 12 omega 6	1.36	1.45	NS	**	**	0.37
C20:0	0.55	0.50	*	**	NS	0.06
ALA, C18:3 omega 3	1.16	1.41	NS	**	**	0.18
C22:0	0.22	0.29	NS	**	**	0.11
C24:0	0.20	0.16	NS	**	*	0.05
C22:6 omega 6	0.16	0.14	NS	NS	NS	0.02
cis-9 trans-11 CLA	0.34	0.63	*	*	**	0.16
trans-10 cis-12 CLA	0.09	0.08	NS	**	**	0.03
SFA	73.29	69.12	**	*	NS	2.52
MUFA	23.44	26.62	**	**	NS	2.09
PUFA	3.29	4.27	**	**	**	0.64
∑ CLA	0.43	0.71	*	*	**	0.14
PUFA omega 3	1.16	1.41	NS	**	**	0.18
PUFA omega 6	1.79	2.22	**	**	**	0.43
C14:1/C14:0	0.033	0.028	**	**	NS	0.01
C16:1/C16:0	0.015	0.013	NS	**	*	0.01
C18:1/C18:0	0.161	0.299	**	*	NS	0.08
cis-9 trans-11 CLA / C18:1 trans-11	0.016	0.030	**	**	**	0.01

SFA, saturated fatty acids; MUFA, monounsaturated fatty acids; PUFA, polyunsaturated fatty acids; CLAs, conjugated linoleic acids. *, *p* < 0.05; **, *p* < 0.01; NS, Not Significant. SEM, standard error of mean.
